# Immunomodulatory Effect of a *Salvia plebeia* R. Aqueous Extract in Forced Swimming Exercise-induced Mice

**DOI:** 10.3390/nu12082260

**Published:** 2020-07-28

**Authors:** Jinseop Shin, Ok-kyung Kim, Shintae Kim, Donghyuck Bae, Jeongmin Lee, Jeongjin Park, Woojin Jun

**Affiliations:** 1 Division of Food and Nutrition, Chonnam National University, Gwangju 61186, Korea; seobseop2@naver.com (J.S.); 20woskxm@chonnam.ac.kr (O.-k.K.); rotoman1@naver.com (S.K.); 2 Jeonnam Bioindustry Foundation, Jeonnam Institute of Natural Resources Research, Jeollanamdo 59338, Korea; bdhyuch@naver.com; 3 Department of Medical Nutrition, Kyung Hee University, Gyeonggido 17104, Korea; jlee2007@khu.ac.kr

**Keywords:** adaptive immunity, forced swimming, innate immunity, immunomodulatory effect, *Salvia plebeia* R.

## Abstract

This study investigated the immunomodulatory effect of *Salvia plebeia* R. aqueous extract (FIE-SP, SPW) in forced swimming exercise-induced mice and the immunostimulatory effects on Raw264.7 cells. Mice were randomly assigned to four groups: the control group (CON), the forced swimming test group (FST), and two FIE-SP groups (low and high dose of FIE-SP). Compared with the control group, the FIE-SP groups showed significantly increased ratios of T lymphocyte surface markers CD4^+^/CD8^+^ and major histocompatibility complex (MHC)I/MHCII, as well as increased concentrations of immunoglobulin (Ig)A and IgG. FIE-SP groups significantly increased Th1 cytokines and decreased Th2 cytokines compared with negative control exercise-induced mice. Conversely, the immunostimulatory effects of FIE-SP significantly increased phagocytic activities, nitric oxide (NO) production, and pro-inflammatory cytokines tumor necrosis factor alpha (TNF-α) and interleukin (IL)-1β in Raw264.7 cells. Furthermore, FIE-SP increased natural killer (NK) cell activities and cytokines (IL-12) in splenocytes compared with the CON group. These results indicated that FIE-SP supplementation could prevent imbalanced immune states and produce immunostimulatory effects to support innate immunity.

## 1. Introduction

Immunity refers to all mechanisms used by the body to protect against foreign environmental agents such as pathogens [1]. Immune mechanisms are composed of various immune cells and cytokine protein molecules. Multiple immune cells regulated by specific cytokines must express specific receptors for cytokines, and cells exposed to cytokines regulate positive or negative host immune responses, depending on the amount and type of exposure [2]. Essential to the immune response are various immune cells and organs that cooperate in protecting the body against infections by pathogens, injuries, and external contaminants [3,4,5,6]. However, immune system imbalances due to internal and external pathogens can lead to diseases caused by autoimmunity, chronic inflammation, and cancer [7]. Other causes of a depressed immune system such as aging, nutritional status, physical stress such as acute exercise, physical disabilities, hyperlipidemia, and adult diseases, including obesity, also affect the host immune system [8]. Strenuous physical activity is associated with increased infection risk [9]. Regular exercise has a positive effect on host immunity [10], but irregular, long-term, one-time, high-intensity exercise results in adverse effects on host immunity [11]. Post-exercise immune function-related depression is most pronounced when exercise is continuous, prolonged (>1.5 h), of moderate to high intensity (55–75% of aerobic capacity), and performed without food intake [12]. Acute exercise depresses many functions of the innate immune system, including phagocytic activity associated with innate immune cells, degranulation of neutrophils, and natural killer cell (NK cell) cytotoxicity [13]. Similarly, other important adaptive immune system functions, including immunoglobulin expression by B lymphocytes [14], imbalanced Th1, and Th2 cytokine production of T lymphocyte differentiation, are reduced after acute exercise [15].

*Salvia plebeia* R. (SP) is a biannual herb that is distributed widely across many countries. In traditional Korean medicine, herbs with fragrant branches are called “*yeojicho*” [16]. SP has a strongly bitter, spicy, and astringent taste. It is considered mainly effective against cough, asthma, and inflammation [17]. The main constituents of SP extracts are mainly flavonoids and phenolic substances, saponins, and essential oils, while the seeds also contain fatty oils [18,19]. In addition, homoplantaginin, nepetin-7-glucoside, luteolin-7 glucoside, hispidulin, eupatorin, and rosmarinic acid have been reported to have anti-inflammatory, anti-oxidant, anti-obesity and hepatocyte protective effects [20,21,22,23,24]. Furthermore, in previous studies, SP extract has been shown to have a variety of biological activities. However, research regarding the immunomodulatory effects in the forced swimming exercise-induced model is insufficient.

Thus, this study aimed to investigate the immunostimulatory effects of aqueous extracts from FIE-SP on murine macrophage Raw264.7 cells, and the immunomodulatory effects in the serum and splenocytes from C57BL/6 mice in the forced swimming model.

## 2. Materials and Methods

### 2.1. Sample and Chemicals

The aqueous extract of *Salvia plebeia* R. (FIE-SP, SPW) was provided by FromBio (Kyounggido, Korea). Briefly, dried leaves of SP were cultivated in Gyeongsangbuk-do, Korea, soaked with water, and the mixture was then heated for 8 h at 80–85 °C, filtered, and dried by a spray dryer. Dulbecco’s modified eagle medium (DMEM), RPMI-1640, fetal bovine serum (FBS), and penicillin were obtained from Gibco BRL (Grand Island, NY, USA). The CyQUANT™ lactate dehydrogenase (LDH) cytotoxicity assay kit and the immunoglobulin ELISA assay kit were purchased from Thermo Fisher Scientific (Waltham, MA, USA). Concanavalin A, IL-1β, IL-2, IL-4, IL-10, IL-12, IFN-γ, and TNF-α ELISA assay kits were purchased from R&D system (Mckinley Place, NE, USA). Mouse CD45 PerCP, mouse CD8a FITC, mouse CD4 PE, mouse major histocompatibility complex (MHCI), FITC, and mouse MHCII PE antibody were purchased from eBioscience (San Diego, CA, USA).

### 2.2. Cell Culture

Mouse murine macrophage cell line Raw264.7 cells and murine leukemia cell line Yac-1 cells were obtained from the American Type Culture Collection (Manassas, VA, USA). Raw264.7 cells were cultured in DMEM supplemented with 10% FBS (inactivated) and 1% penicillin/streptomycin. Yac-1 cells were cultured in RPMI 1640 supplemented with 10% FBS and 1% penicillin/streptomycin and maintained in a 75T flask at 37 °C under a humidified atmosphere of 5% CO_2_.

### 2.3. Animals

Four-week-old C57BL/6 mice were obtained from Orient Bio Inc. (Kyounggido, Korea). All mice underwent 1 week of acclimation before experiments were performed. The animals were housed in stainless steel cages in an air-conditioned room with controlled temperature (22–25 °C) and automatic lighting (alternating 12 h periods of light and dark). They all received 5L79 diet (Orient Bio Inc., Kyounggido, Korea) and water ad libitum. All studies were performed under the Guide for Animal Experimentation of Chonnam National University and approved by the Institutional Animal Care and Use Committee of Chonnam National University (CNU IACUC-YB-2019-86).

Forty mice were randomly divided into four groups: CON (untreated control), forced swimming control (FSC), FIE-SP-L (FIE-SP 200 mg/kg), and FIE-SP-H (FIE-SP 600 mg/kg). CON and FSC were orally administered water, while the FIE-SP-L and FIE-SP-H group mice were orally administered FIE-SP at 200 mg/kg/day and 600 mg/kg/day, respectively, for the experimental duration.

### 2.4. Forced Swimming Test

We used the mouse forced swimming test to evaluate the effect of FIE-SP treatment on immune dysfunction. In brief, the apparatus used in this test was an acrylic plastic pool (width, depth, and length (cm): 90 × 45 × 45). The pool was filled with water to a depth of 38 cm. The temperature of the water was maintained at 34 ± 1 °C. The pool current was generated by circulating the water with a pump. The swimming pool flow rate was 6 L/min. Before the end of the 2-week treatment, including the FSC group, FIE-SP-L, and FIE-SP-H groups, a 3-day continuous forced swimming test was performed. Forced swimming was defined as the time from placement of the mouse into the pool until the time that the animal remained submerged for 7 sec without movement or rising to the water surface to breathe.

### 2.5. Flow Cytometry

Cell staining was performed at 4 °C in phosphate-buffered saline (PBS) supplemented with 1% FBS unless otherwise indicated. Antibodies and reagents used for flow cytometry and functional studies were as follows: mouse CD45 PerCP, mouse CD8a FITC, mouse CD4 PE, mouse MHCI FITC, mouse MHCII PE (eBioscience, San Diego, CA, USA). Data were collected using a CytoFLEX flow cytometer (Beckman Coulter) and analyzed using CytoExpert.

### 2.6. Measurement of Immunoglobulin in the Serum

Serum was collected for analysis from mice used in the forced swimming test. Serum IgG and IgA were analyzed using ELISA kits according to the manufacturer’s instructions.

### 2.7. Measurement of Cytokine Production

Raw264.7 cells were cultured in 96-well plates at a density of 1 × 10^6^ cells/well, incubated for 24 h with various concentration of FIE-SP (50, 100, 150 or 200 µg/mL) and lipopolysaccharides (LPS) (1 µg/mL). The supernatant was collected for analysis, after incubation at 37 °C in a 5% CO_2_ incubator for 24 h, for TNF-α and IL-1β. Splenocytes isolated from the C57BL/6 mice were cultured in 96-well plates at a density of 1 × 10^6^ cells/well with T cell mitogen Con A 5 μg/mL and incubated for 24 h. The supernatant was collected for analysis, after incubation at 37 °C in a 5% CO_2_ incubator for 24 h (IL-2), 48 h (IL-4, IL-10, and IL-12), and 72 h (IFN-γ). Supernatant cytokines were quantified using a Duoset sandwich ELISA Mouse kit according to the manufacturer’s instructions.

### 2.8. Cytotoxicity in Raw264.7 Cells

Cell viability was measured using the 2,3-Bis(2-methoxy-4-nitro-5-sulfophenyl)-2*H*-tetrazolium-5-carboxanilide inner salt or XTT assay. The staining reagent contained 100 μM of phenazine methosulfate (PMS) in PBS and 1 mg/mL XTT in phenol red-free DMEM (PMS: XTT = 1:800). The cells were cultured and incubated for 24 h. After the incubation, the cells were treated with increasing concentrations of FIE-SP (0–1000 μg/mL) for 24 h. Subsequently, the staining reagent was added to each well and incubation continued for 2 h at 37 °C. Cell viability was assessed by measuring the absorbance at 450 nm.

### 2.9. Phagocytosis Activity in RAW264.7 Cells

Raw264.7 macrophages were pretreated for 3 h with FIE-SP (50, 100, 150 or 200 µg/mL) and LPS (1 µg/mL). Nonopsonized zymosan particles (10 μL/sample) were added and incubated for 2 h. The amount of engulfed zymosan particles was determined using CytoSelect 96-well phagocytosis assay from Cell Biolabs (San Diego, CA, USA). The absorbance was measured at 405 nm using a 96-well microplate reader (BioTek Inc., Winooski, VT, USA).

### 2.10. Measurement of NK Cell Activity

Splenocytes isolated from the spleen were cultured in 96-well plates at a density of 1 × 10^5^ cells/well and co-cultured with 1 × 10^4^ Yac-1 cells/well. The ratio of effector cells and target cells was 10:1 and the plates were incubated at 37 °C in a 5% CO_2_ incubator for 4 h. After incubation, 50 µL of supernatant was dispensed into a new 96-well plate, using a CyQUANT™ LDH cytotoxicity assay kit according to the manufacturer’s instructions.

### 2.11. Statistical Analysis

All data are presented as the mean ±S.D. or ±S.E. Statistical analysis was performed using one-way ANOVA with Student’s *t*-test for two-sample comparisons. SPSS (Statistical package for social science version 23.0, SPSS Inc., Chicago, USA) was used for statistical calculations, and *p* < 0.05 was considered significant.

## 3. Results

### 3.1. Effect of FIE-SP on CD4/CD8 and MHCI/MHCII Ratio in Forced Exercise-Induced Mice

**Figure 1:**
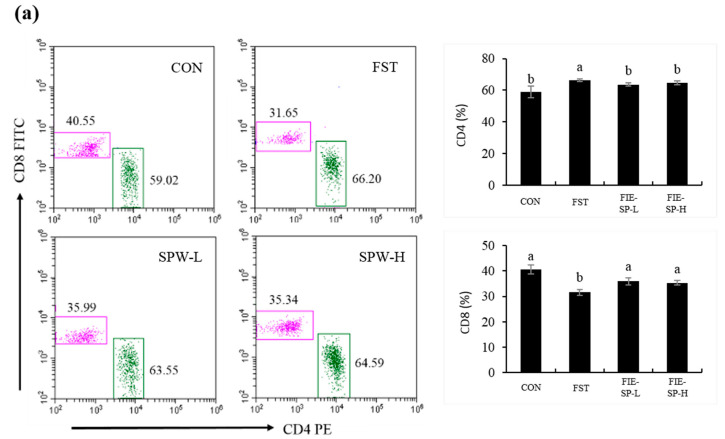

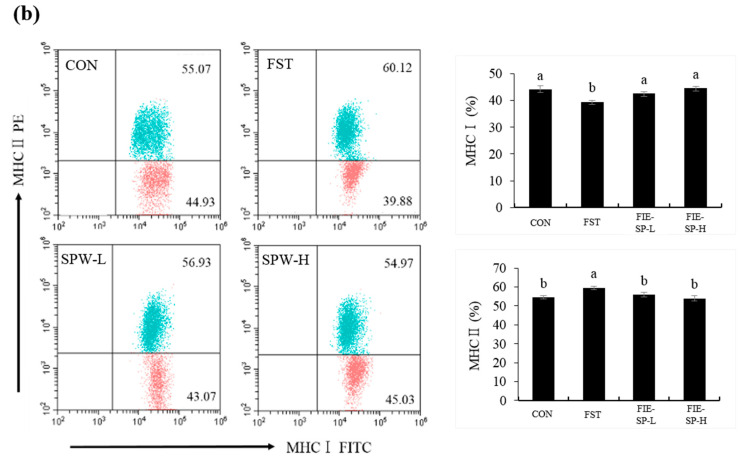
The ratio of CD4/CD8, MHCI/MHCII in C57BL/6 mouse splenocytes following oral administration of a *Salvia plebeia* extract was determined using flow cytometry. (**a**) The CD4^+^ and CD8^+^ T lymphocyte ratio. (**b**) The MHCI and MHCII ratio. Data shown are representative of four experimental groups: CON, the control group; FST, the forced swimming test group; FIE-SP-L, the low-concentration *Salvia plebeia* R. extract-treated group; FIE-SP-H, the high-concentration *Salvia plebeia* R. extract-treated group. Data are expressed as the mean ± S.E. Different letters in a column indicate statistical differences based upon Duncan’s multiple range test (*p* < 0.05). CON = control group; FST = forced swimming test group; SPW-L = low concentration *Salvia plebeia* R. extract; SPW-H = high-concentration *Salvia plebeia* R. extract.

To evaluate the potential immunomodulatory activity of FIE-SP, the frequency of immune cell populations was measured using flow cytometry (Figure 1). Compared with the control group, treatment with FIE-SP groups significantly increased the frequencies of CD4^+^ and MHCII populations in splenocytes. Correspondingly, the CD8^+^ and MHCI frequency was significantly decreased in the forced exercise group.

### 3.2. Effect of FIE-SP on Serum Immunoglobulin in Forced Exercise-Induced Mice

**Figure 2:**
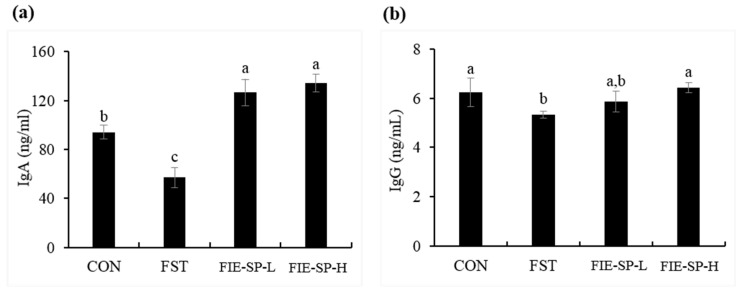
The level of immunoglobulin in C57BL/6 mouse serum treated with *Salvia plebeia* extract was determined using ELISA. (**a**) Immunoglobulin A (IgA). (**b**) Immunoglobulin G (IgG). Data are expressed as the mean ± S.E. Different letters in a column indicate statistical differences based upon Duncan’s multiple range test (*p* < 0.05).

As shown in the representative results for the immunoglobulin levels of each experimental group (Figure 2), we determined that the two immunoglobulin concentrations were significantly decreased in the forced exercise group compared with the control group, and the levels increased in the FIE-SP administration groups.

### 3.3. Effect of FIE-SP on Th1/Th2 Cytokine Production in Forced Exercise-Induced Mice

**Figure 3:**
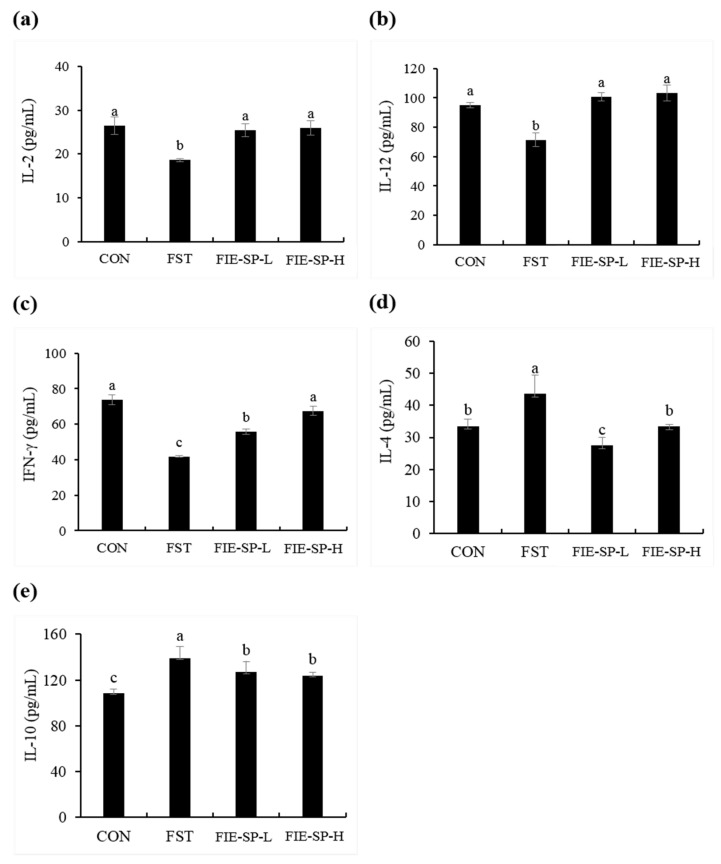
Effect of *Salvia plebeia* extract on the levels of C57BL/6 mouse splenocyte cytokines in the culture medium. Levels of Th1 cytokines (**a**) Interleukin-2 (IL-2), (**b**) IL-12, and (**c**) Interferon-γ (IFN-γ) and Th2 cytokines (**d**) IL-4 and (**e**) IL-10 were determined using ELISA. Data are expressed as the mean ± S.E. Different letters in a column indicate statistical differences based upon Duncan’s multiple range test (*p* < 0.05).

Next, we assessed whether oral administration of FIE-SP in C57BL/6 mice altered splenocyte cytokine levels following forced exercise. As shown in Figure 3, we observed that the Th1 cytokines (IL-2, IL-12, and IFN-γ) were significantly decreased in the FST group compared with the CON group. The FIE-SP oral administration group showed a significant increase in Th1 cytokines compared with the forced exercise group. We also measured Th2 cytokines (IL-4 and IL-10). Contrary to the results for Th1 cytokine production, the IL-4 and IL-10 production was significantly increased in the FST group.

### 3.4. Effect of FIE-SP on Macrophage Activities in Raw264.7 Cells

**Figure 4:**
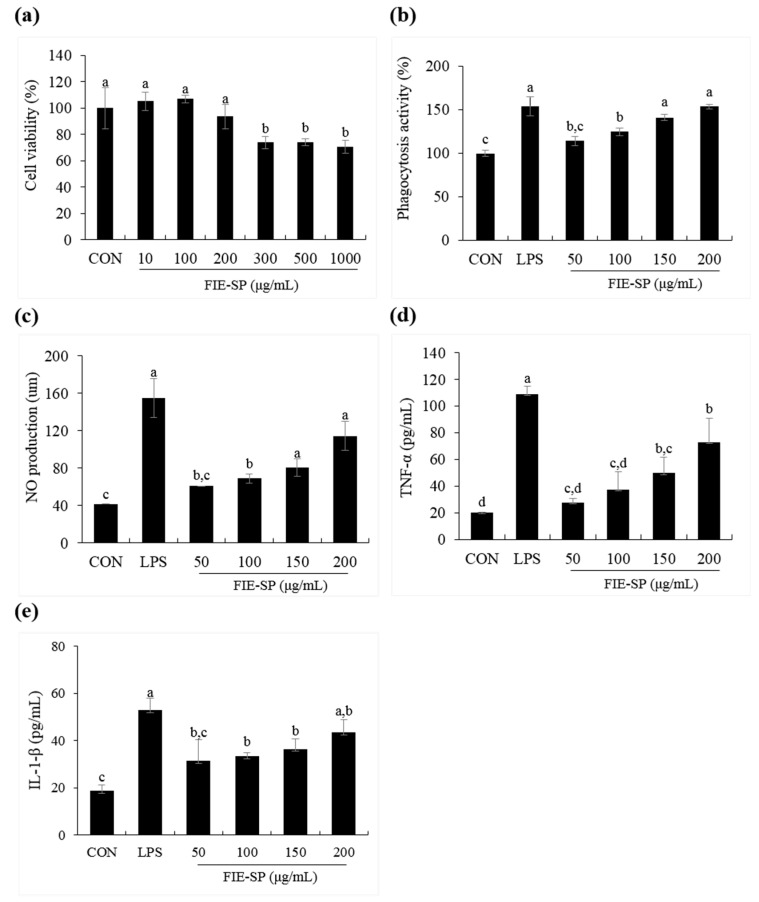
Macrophage stimulatory effects of FIE-SP in Raw264.7 cells. (**a**) Viability of Raw264.7 cells following treatment with various concentrations of FIE-SP. (**b**) Phagocytosis in FIE-SP-treated Raw264.7 cells. (**c**) NO production in FIE-SP-treated Raw264.7 cells. Pro-inflammatory cytokine secretion in FIE-SP-treated Raw264.7 cells. (**d**) Tumor necrosis factor-α (TNF-α) and (**e**) IL-1β secretion. LPS, 1 µg/mL. Data are expressed as the mean ±S.D. Different letters in a column indicate statistical differences based upon Duncan’s multiple range test (*p* < 0.05).

We measured macrophage stimulatory activity to investigate the effect of FIE-SP on the stimulation of innate immunity as well as adaptive immunity (Figure 4). We evaluated the levels of macrophage stimulating factors such as phagocytosis activity, NO, TNF-α, and IL-1β compared with the control and FIE-SP-treated groups. Cytotoxicity was absent following FIE-SP treatments up to 200 µg/mL. FIE-SP significantly increased phagocytosis activity at concentrations of 100 µg/mL. Similarly, NO, TNF-α, and IL-1β increased following FIE-SP extract treatment in a dose-dependent manner.

### 3.5. Effect of FIE-SP on NK Cell Activities in Splenocytes

**Figure 5:**
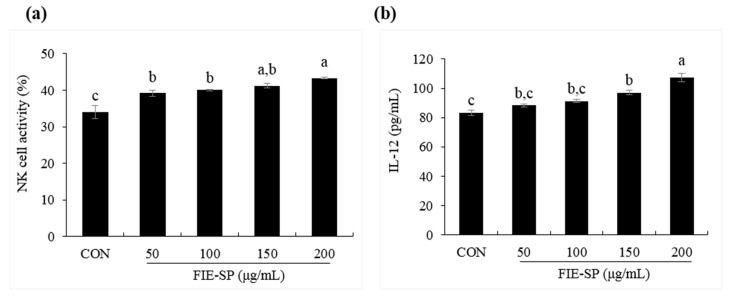
Natural killer (NK) cell activity following FIE-SP treatment of splenocytes. (**a**) Various concentrations of NK cell activities of splenocytes. (**b**) IL-12 cytokine secretion in FIE-SP-treated splenocytes. Data are expressed as the mean ±S.D. Different letters in a column indicate statistical differences based upon Duncan’s multiple range test (*p* < 0.05).

Finally, we investigated whether the immune-stimulating effects of FIE-SP treatment correlated with NK cell activity and cytokine changes in splenocytes (Figure 5). Compared with the control, FIE-SP treatment significantly increased lactate dehydrogenase (LDH) and IL-12 level from splenocytes. Thus, FIE-SP was expected to have effective potential functions in the innate immune system by increasing NK cell activity.

## 4. Discussion

The immune system is divided into the innate and the adaptive immune system. The balance of the two different immune systems must correspond to each other. Strenuous physical activity is associated with increased infection risk [12]. Exercise-related immunological changes include signs of inflammation, such as the release of cytokines, activation of immunocompetent cell lines and complements, and the induction of acute-phase proteins [25,26,27,28]. Furthermore, strenuous exercise increases the signs of immunosuppression [29,30], such as decreased T and B cell function [31] and impaired cytotoxic or phagocytic activity [15]. The immunological response to exercise comprises numerous alterations within the immune system, but how these processes are regulated is still largely unknown.

During the 14-day oral administration of FIE-SP, forced swimming exercise was performed once a day for three days before sacrifice. In our previous studies, we demonstrated that NK cell activity and Th1-related cytokines (IL-2, IL-12 and IFN-γ) were significantly decreased in the exercise group that performed forced swimming exercise for 3 days in comparison with the non-exercise control group (data not shown). Therefore, we determined that three days of forced swimming exercise imbalances immunity.

High-intensity exercise is a type of physiological stress that has effects on immune cell life spans. Strenuous exercise decreases the activity of various immune cells and induces lymphocyte apoptosis due to increased reactive oxygen species (ROS) [32,33,34]. Previous research showed a decreased ratio of CD4^+^/CD8^+^ after exercising for 15, 45, 60, and 120 min [35,36,37,38]. Increased apoptosis was observed in humans after endurance exercises such as running a marathon [39] or high-intensity treadmill running [40]. In our study, CD45 is required to aid in the identification of lymphocytes in splenocyte. Therefore, we first isolated, by flow cytometry analysis, a T lymphocyte singlet through CD45 marker staining before counting the CD4^+^/CD8^+^ ratio, which is a subset of T lymphocytes, using splenocytes isolated from C57BL/6 mice. The ratio of lymphocyte subsets and the surface markers for the proportion of CD4^+^ T cells and MHCII increased in the exercise group compared with the control group and decreased in the FIE-treated groups. As a result, our research determined that forced swimming exercise acted as physiological stress and induced an imbalance of T lymphocytes and the surface marker ratio due to the increased lymphocyte apoptosis mediated by accumulated ROS. Thus, FIE-SP extract might be effective in balancing T lymphocytes and surface markers.

Based on the above results, we evaluated changes in cytokines between groups due to forced swimming exercise in Th1 and Th2-related cytokines, a phenotype of CD4^+^ T cells, and evaluated specific changes in FIE-SP-treated groups compared with FST groups. Our results showed that Th2-cytokines were significantly increased in the FST group. Previous research showed that exhausting exercise, such as marathon running, has an immunosuppressive effect that induces increased Th2 cytokines and inhibits Th1 cytokines [41]. Th2-type cytokines such as IL-4, when increased relative to Th1 cytokines (IFN-γ, IL-2), cause asthma and exercise-induced allergy and induce disease vulnerability [42]. As a result, we determined that the ratio of Th1 to Th2-related cytokines was decreased by forced exercise, and Th2 was produced more than Th1 due to vigorous physical activity, causing immunosuppression. FIE-SP might be useful in balancing Th1 and Th2 cytokines in an immunosuppression model induced by exhausting exercise.

B cells induce humoral immune responses by producing antibodies called immunoglobulins against specific invading antigens [2]. Previous studies have shown that nasal and salivary IgA concentrations are reduced after heavy exertion [43]. We measured IgA and IgG, the immunoglobulins involved in the immune response. The results showed that the amount of immunoglobulin was significantly decreased in the FST group compared with the CON group without exercise and increased in the FIE-SP-treated groups. As a result, our study showed a decrease in serum immunoglobulins in the immunosuppressive state due to high-intensity exercise, and FIE-SP may be useful in restoring immunoglobulins in an exercise-induced immunosuppression model.

We further examined the macrophage cell line Raw264.7 and spleen cells using an in vitro assay to investigate the effect of FIE-SP on the stimulation of innate immunity. Previous studies have shown that TNF-α and IL-6 secretion was increased in spleen cells treated with *Salvia plebeia* R. [15]. Similarly, our experimental results on the macrophage stimulation effect from various concentrations of FIE-SP were compared with the LPS (1 µg/mL) stimulated group and the untreated group. First, phagocytosis activity of Raw264.7 cells was higher after FIE-SP treatment than that in the control group. The expression level of pro-inflammatory cytokines increased with the concentration of FIE-SP treatment. In addition, NO production was also higher following FIE-SP treatment than that in the control group. This finding suggests that FIE-SP might activate macrophages and have potential effects on innate immunity. We also cultured splenocytes isolated from C57BL/6 mice with FIE-SP to measure NK cell activity and the NK-related cytokine IL-12. As a result of NK cell activity, the amount of LDH secretion in the FIE-SP-treated group was higher than that in the control group. IL-12 also showed higher secretion in the FIE-SP group than in the control group. This finding suggests that FIE-SP extract has a variety of effects on NK cells involved in endogenous immunity, resulting in increased cytotoxicity for YAC-1 cells and the same effects on IL-12, which affect NK cell activation.

Together, our research demonstrated not only the effect of FIE-SP extract immunostimulation in Raw264.7 cells, which is of considerable significance to innate immunity, but also the immunomodulatory effects of forced swimming exercise-induced mice. Overall, the results of this study suggest that FIE-SP enhances immunity by improving various immune cell functions, and these findings may broaden the application of natural product-based immune therapy.

## 5. Conclusions

In conclusion, our study reports that *S. plebeia* is effective in stimulation of innate immune cell activity and prevention of immune dysfunction according to the forced swimming exercise-induced immunosuppression model. It is presumed that the mechanism of *S. plebeia* against immune stimulation might involve the modulation of T lymphocyte surface marker and innate immune cell activity. The results suggest that *S. plebeia* in particular might be developed as an immunostimulant.
